# The role of conserved residues in Fdc decarboxylase in prenylated flavin mononucleotide oxidative maturation, cofactor isomerization, and catalysis

**DOI:** 10.1074/jbc.RA117.000881

**Published:** 2017-12-19

**Authors:** Samuel S. Bailey, Karl A. P. Payne, Karl Fisher, Stephen A. Marshall, Matthew J. Cliff, Reynard Spiess, David A. Parker, Stephen E. J. Rigby, David Leys

**Affiliations:** From the ‡ Manchester Institute of Biotechnology, University of Manchester, 131 Princess Street, M1 7DN, United Kingdom and; § Innovation/Biodomain, Shell International Exploration and Production, Westhollow Technology Center, Houston, Texas 77082-3101

**Keywords:** flavin, enzyme catalysis, enzyme mechanism, oxidation-reduction (redox), decarboxylase, radical, enzyme structure, crystal structure, EPR, prenylated flavin mononucleotide (prFMN), UbiD

## Abstract

The UbiD family of reversible decarboxylases act on aromatic, heteroaromatic, and unsaturated aliphatic acids and utilize a prenylated flavin mononucleotide (prFMN) as cofactor, bound adjacent to a conserved Glu–Arg–Glu/Asp ionic network in the enzyme's active site. It is proposed that UbiD activation requires oxidative maturation of the cofactor, for which two distinct isomers, prFMN^ketimine^ and prFMN^iminium^, have been observed. It also has been suggested that only the prFMN^iminium^ form is relevant to catalysis, which requires transient cycloaddition between substrate and cofactor. Using *Aspergillus niger* Fdc1 as a model system, we reveal that isomerization of prFMN^iminium^ to prFMN^ketimine^ is a light-dependent process that is largely independent of the Glu^277^–Arg^173^–Glu^282^ network and accompanied by irreversible loss of activity. On the other hand, efficient catalysis was highly dependent on an intact Glu–Arg–Glu network, as only Glu → Asp substitutions retain activity. Surprisingly, oxidative maturation to form the prFMN^iminium^ species is severely affected only for the R173A variant. In summary, the unusual irreversible isomerization of prFMN is light-dependent and probably proceeds via high-energy intermediates but is independent of the Glu–Arg–Glu network. Our results from mutagenesis, crystallographic, spectroscopic, and kinetic experiments indicate a clear role for the Glu–Arg–Glu network in both catalysis and oxidative maturation.

## Introduction

A wide range of enzymes are known to catalyze decarboxylation, many requiring cofactors, such as PLP, metal ions, or flavin, for catalytic activity (1, 2). The flavins FMN and FAD represent arguably one of the most versatile cofactors, responsible not only for a range of redox reactions, but also for light-dependent catalysis (3, 4). The chemical properties of a flavin are influenced by the microenvironment inside the enzyme active site and are occasionally altered by covalent modification at the C6 or C8 position of the isoalloxazine dimethylbenzene moiety (5). Recently, a highly modified form of flavin, prenylated FMN (prFMN),^2^ incorporated by decarboxylases belonging to the UbiD family of enzymes was described (6–8). The UbiD superfamily is composed of a wide variety of (de)carboxylases acting on aromatic, hetero-aromatic, and unsaturated aliphatic acids (9). Prenylated FMN consists of an FMN molecule modified by the addition of a fourth non-aromatic ring joined via N5–C1′ and C6–C3′ linkages between the flavin and prenyl moieties.

**Figure 1.**
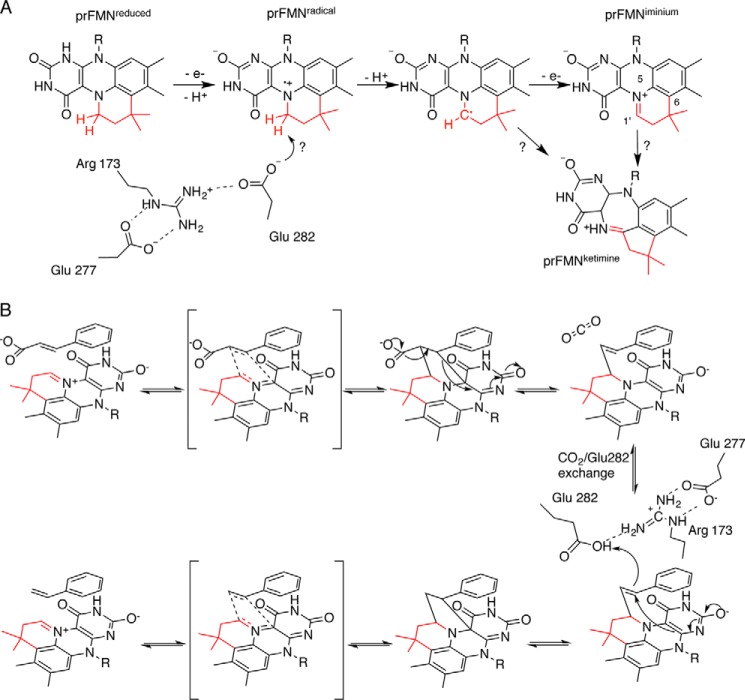
**Overview of prFMN maturation and catalysis in Fdc1.** *A*, a schematic representation of the stepwise oxidation of reduced prFMN, which might involve deprotonation by Glu^282^ in Fdc1 to form the iminium form. How the ketimine species is formed is unclear. *B*, proposed mechanism for Fdc1 catalysis involving 1,3-dipolar addition between the substrate and the prFMN^iminium^ azomethine ylide, leading to a covalent substrate-prFMN^iminium^ pyrrolidine adduct. Fragmentative decarboxylation is coupled to the breaking of the β-carbon–prFMN C4a bond. The protonation of the substrate-cofactor complex by Glu^282^, concurrent with formation of a second pyrrolidine adduct, leads to product release via a retro-1,3-dipolar cycloaddition.

The enzyme responsible for the biosynthesis of prFMN is UbiX (6), which forms the fourth ring via the addition of the isoprene moiety of dimethylallyl-monophosphate to FMNH_2_. It is proposed that prFMN is released from UbiX and bound by apo-UbiD enzymes in a reduced form (prFMN^reduced^). Following binding of prFMN^reduced^ and formation of holo-UbiD, the cofactor undergoes oxidation to form the catalytically relevant oxidized prFMN species (Fig. 1*A*).

Surprisingly, atomic resolution crystal structures of *Aspergillus niger* Fdc1, a UbiD-type (de)carboxylase, revealed two forms of the oxidized cofactor. These correspond to an isoalloxazine *N*^5^-iminium form (prFMN^iminium^) and the isomeric *N*^5^-secondary ketimine form (prFMN^ketimine^), the latter having a very distinct ring structure derived from the isoalloxazine ring system (Fig. 1*A*). Although a putative mechanism for decarboxylation has been postulated for both of these forms, there are several indications that prFMN^iminium^ is the catalytically relevant form (7, 10, 11). Mechanistic insights have been obtained from the structure of a covalent substrate–cofactor adduct of *A. niger* Fdc1 with α-hydroxycinnamic acid, a close mimic of the natural substrate cinnamic acid. This adduct reveals a covalent bond between the prenyl-C1′ of the prFMN^iminium^ cofactor and a molecular species derived from the decarboxylation of α-hydroxycinnamic acid. Combined with solution data, this led to the suggestion that prFMN^iminium^ catalyzes substrate (de)carboxylation via formation of a covalent prFMN-substrate cycloadduct through 1,3-dipolar cycloaddition (Fig. 1*B*). Indeed, the prFMN^iminium^ cofactor has azomethine ylide characteristics (*i.e.* the dipole), whereas many of the UbiD substrates can be classified as dipolarophiles. Isotope effect experiments (10) and theoretical studies (11) have also suggested that prFMN^iminium^ is responsible for 1,3-dipolar cycloaddition–based catalysis. More recently, a mechanism-based inhibitor, 2-fluoro-2-nitrovinylbenzene, was used to detect a cycloadduct via MS, adding to the growing body of evidence for 1,3-dipolar cycloaddition in UbiD enzymes (12). The question remains how the prFMN^ketimine^ is formed and whether it can play any catalytic role.

To what extent the UbiD active site contributes to oxidative maturation, cofactor isomerization, and catalysis is also unclear. In the UbiD enzyme family, Arg^173^, Glu^277^, and Glu^282^ (*A. niger* Fdc1 numbering) form a conserved ionic network. Crystal structures of *A. niger* Fdc1 reveal that Glu^282^ is positioned close to the prFMN C1′ and is therefore most likely to act as the key acid-base. Unlike Arg^173^ and Glu^277^, the Glu^282^ side chain can occupy distinct positions, effectively competing with the substrate carboxylate group or CO_2_ for a site adjacent to Arg^173^. This suggests that Arg^173^ is essential for substrate binding. The role of Glu^277^, which is located in the periphery of the active site, is less clear. Preliminary studies of R173A, E277Q, and E282Q variants suggested that all are catalytically inactive and possess altered UV-visible spectra (7). This implies the role of this network could be 2-fold: first to facilitate oxidative maturation of the cofactor to the iminium form (Fig. 1*A*) and second to act as a key acid-base during catalysis (Fig. 1*B*).

To further elucidate the roles of these residues, we created an additional three Fdc1 variants (R173K, E277D, and E282D) and assessed the effect of each of the six mutations on cofactor maturation and substrate decarboxylation for both the *A. niger* and *Saccharomyces cerevisiae* Fdc1 enzymes. We describe crystal structures and activity measurements of Fdc1 and the variants that demonstrate the light-dependent isomerization of prFMN^iminium^ to prFMN^ketimine^. In addition to the high-resolution crystal structures, mass spectrometric, spectroscopic, kinetic, and hydrogen/deuterium exchange studies of six Fdc1 variants provide clear evidence for the role of the Glu–Arg–Glu motif in both oxidative maturation and catalysis.

## Results

### Light-dependent cofactor isomerization and enzyme inactivation

**Figure 2.**
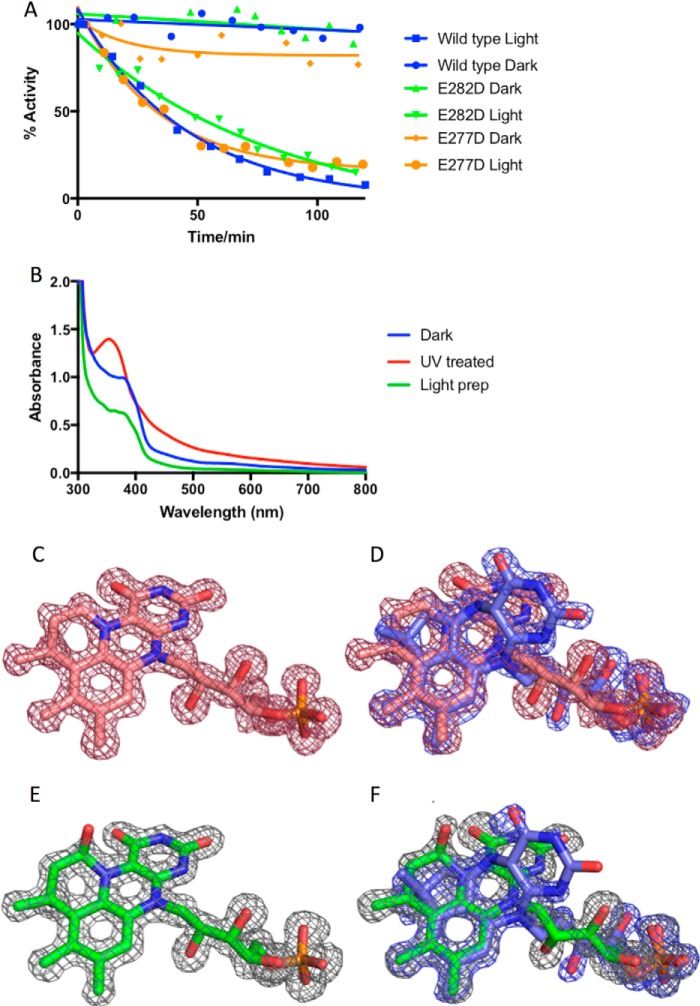
**Effect of illumination on *A. niger* Fdc1 activity.** *A*, a plot of activity over time for *A. niger* Fdc1 wildtype and variants in the presence and absence of light exposure. Concentration of wildtype Fdc1 in the assay was 45 nm. Concentration of E282D and E277D Fdc1 in the assay was 600 and 300 nm, respectively. *B*, UV-visible spectra of *A. niger* wildtype Fdc1 purified in the dark and following illumination with 365-nm UV light. For comparison, the spectrum of protein purified under normal ambient light exposure conditions is also shown. *C*, electron density corresponding to the prFMN cofactor from wildtype *A. niger* Fdc1 that has been purified and crystallized in the dark. Shown is an omit map corresponding to prFMN^iminium^ contoured to 3σ. *D*, electron density of the prFMN cofactor for wildtype *A. niger* Fdc1 following illumination with UV light. Shown is an omit map corresponding to prFMN^iminium^ (in *red*) and prFMN^ketimine^ (in *blue*) contoured to 3σ. *E*, electron density corresponding to the prFMN cofactor for the E282Q *A. niger* Fdc1 purified and crystallized in the dark. Shown is an omit map corresponding to a mixture of prFMN^iminium^/prFMN^hydroxylated^ contoured to 3σ. *F*, electron density of the prFMN cofactor for E282Q *A. niger* Fdc1 crystals illuminated with UV light. Shown is an omit map corresponding to prFMN^hydroxylated^ (in *gray*) and prFMN^ketimine^ (in *blue*) contoured to 3σ.

Following purification, incubation of wildtype holo-Fdc1 on ice leads to loss of decarboxylase activity with a half-life of ∼30 min (Fig. 2*A*). The distantly related AroY also exhibits rapid loss of activity, but only when incubated under aerobic conditions (13). However, when Fdc1 is incubated in the dark, activity remains constant for many hours, even under aerobic conditions. Hence, we repeated the purification of Fdc1 in the dark, revealing that the latter exhibits slightly higher activity (*k*_cat_ = 9.3 ± 0.1 s^−1^) compared with the protein purified under ambient light (*k*_cat_ = 7.6 ± 0.2 s^−1^). The respective enzyme preparations exhibit different UV-visible spectra (Fig. 2*B*), with the “dark” protein preparation displaying a prominent feature at 380 nm and showing subtle differences between 340 and 385 nm compared with protein purified under normal light conditions. Exposure of the dark protein preparation to 365-nm UV light from an LED source for 5 min resulted in complete loss of activity and a change in the UV-visible spectrum (Fig. 2*B*).

High-resolution crystal structures of Fdc1 purified and crystallized in the dark reveal that the prFMN cofactor is exclusively in the iminium form (Fig. 2*C*). Following brief exposure of these crystals to UV light, the corresponding crystal structure reveals isomerization of at least 50% of the prFMN cofactor to the previously observed ketimine form (Fig. 2*D*). Returning the illuminated Fdc1 crystals to the dark for an extended period of time does not revert the prFMN^ketimine^ to prFMN^iminium^, indicating that the isomerization is irreversible. As illumination leads to inactivation, these observations directly confirm that prFMN^iminium^, rather than prFMN^ketimine^, is the active form of the cofactor.

### UV-visible spectrophotometric characterization of Fdc1 Glu^277^ and Glu^282^ variants reveals minor variation in prFMN incorporation and maturation

**Figure 3.**
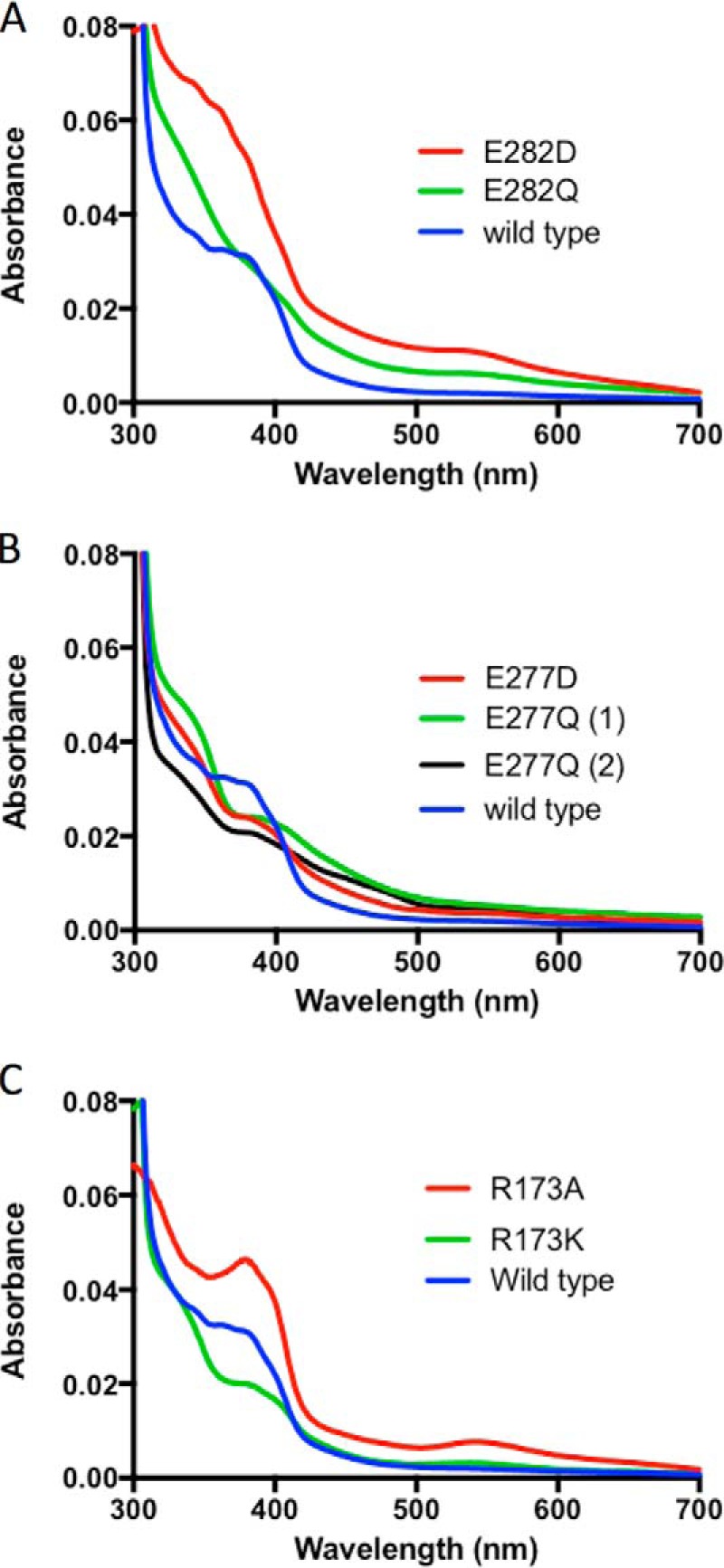
**UV-visible spectra for *A. niger* Fdc1 variants.** Shown is an overlay of individual UV-visible spectra for each of the three conserved active-site residue variants: Glu^282^ (*A*), Glu^277^ (*B*), and Arg^173^ (*C*). Spectra have been normalized for protein concentration using *A*_280_.

Four Fdc1 variants (E277D, E277Q, E282D, and E282Q) were successfully co-expressed with UbiX to produce the corresponding holo-enzymes. The UV-visible spectrum of each variant indicated the presence of prFMN, as indicated by broad absorbance features between 340 and 390 nm and at 550 nm (Fig. 3). However, the exact spectral properties showed subtle variation between the various species. Whereas E282D shows similar features between 340 and 390 nm as the wildtype enzyme, there is also evidence for the presence of an additional minor species, as indicated by the weak feature at 550 nm. The latter has been shown to correspond to an inactive radical form of the cofactor (7). In the case of E282Q, the level of incorporation appears lower than in the wildtype and other variants.

**Figure 4.**
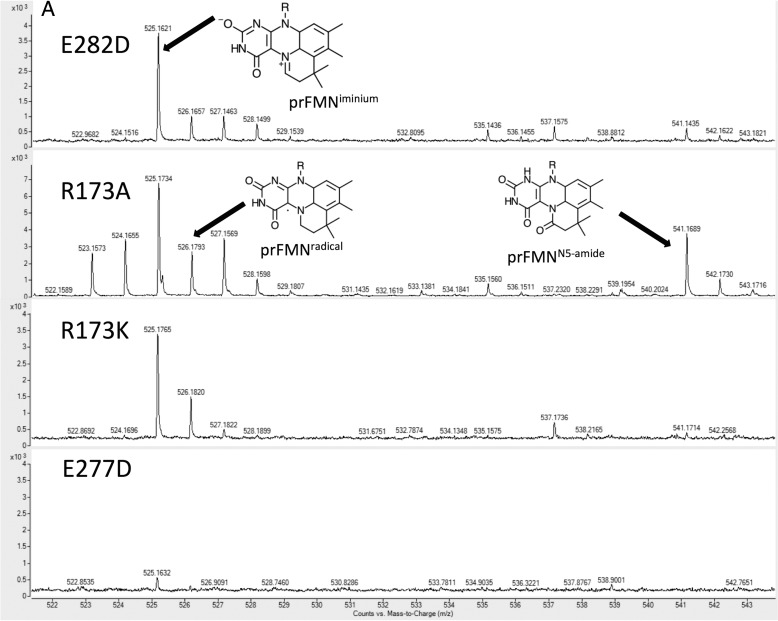

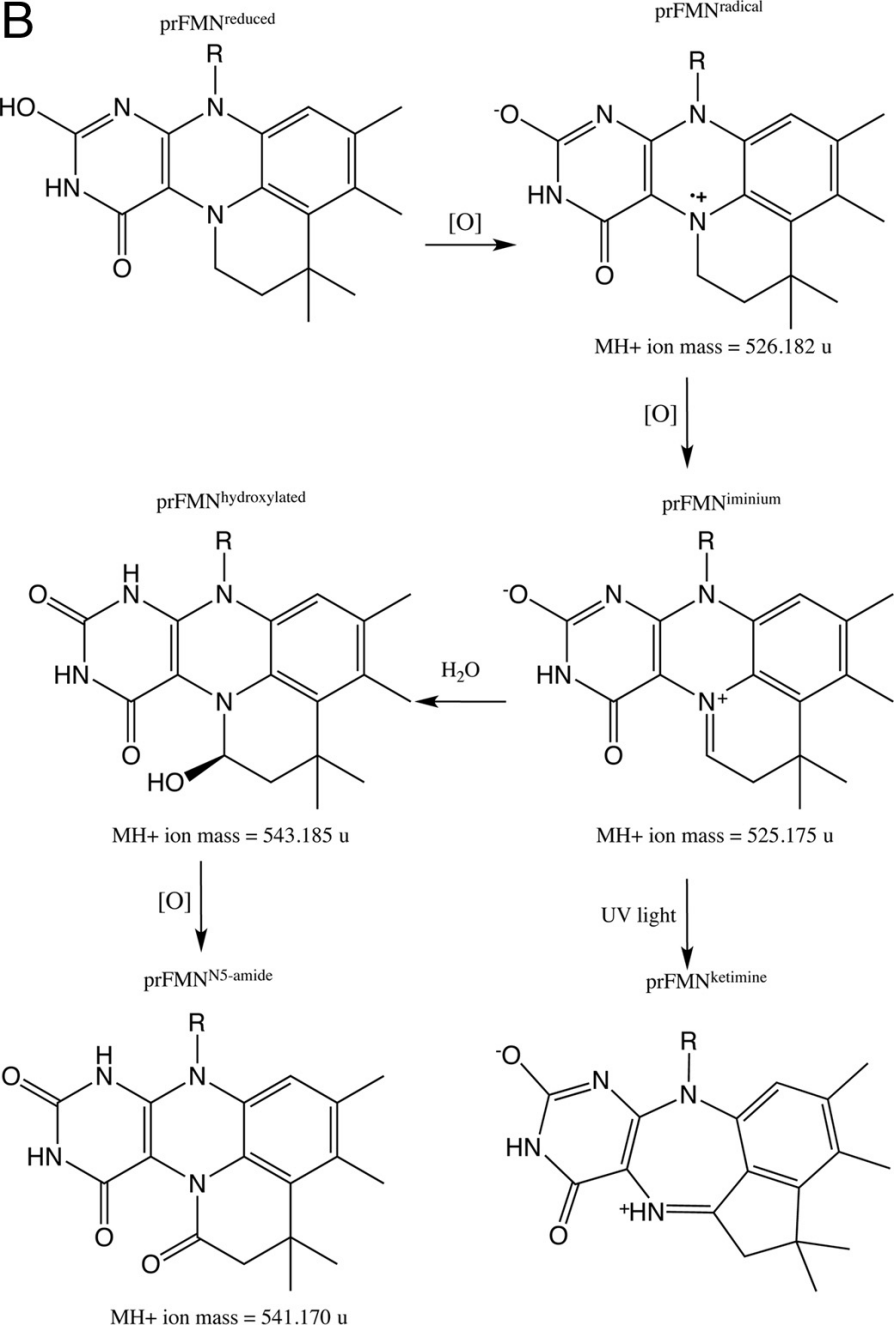
**Mass spectrometry of prFMN extracted from *A. niger* Fdc1 variants.** *A*, a side-by-side comparison of ESI-MS spectra of prFMN extracted from *A. niger* Fdc1 variants. *B*, a proposed scheme for the maturation of prFMN in Fdc1. Species prFMN^radical^, prFMN^iminium^, and prFMN^N5-amide^ have been identified in ESI-MS spectra presented in *A*.

ESI-MS was used to confirm prFMN incorporation, and ESI-MS of both E277D and E282D shows a main MH^+^ ion mass of 525.16 Da, corresponding to prFMN^iminium^ (Fig. 4). Unfortunately, no mass corresponding to the bound prFMN could be observed for either the E277Q or E282Q variant.

### An acidic residue at both position 277 and 282 is required for activity

**Figure 5.**
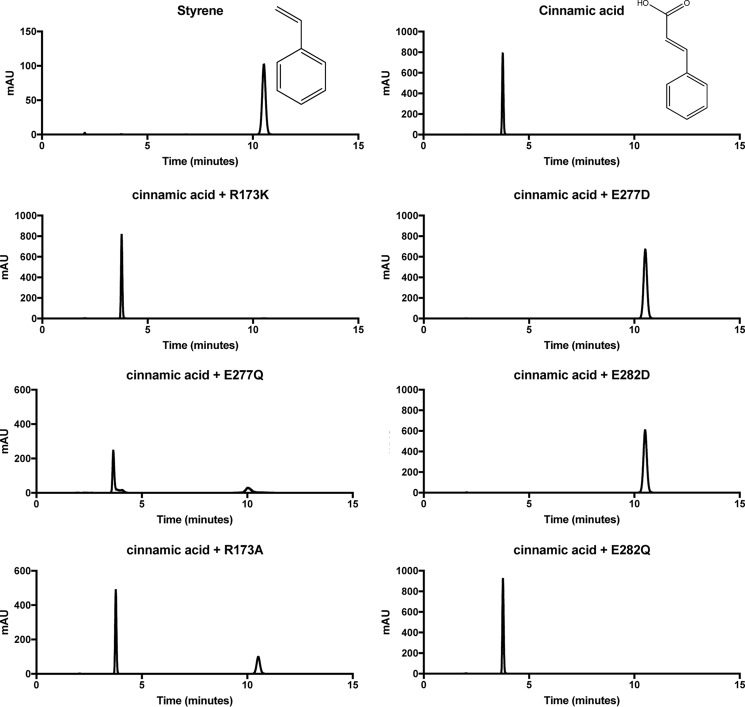
**Detection of decarboxylation activity using HPLC.** Shown is an HPLC chromatogram of a 10 mm cinnamic acid solution in the presence of 50 μm Fdc1 variants.

**Figure 6.**
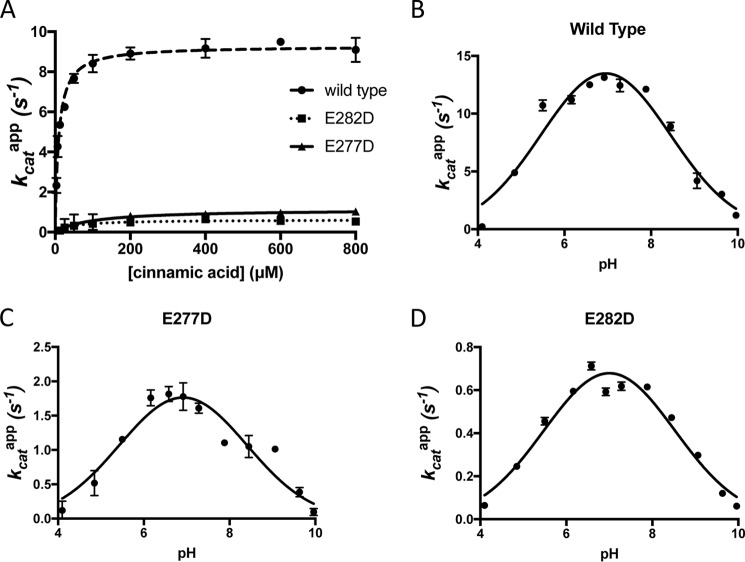
**Characterization of the active variants E282D and E277D.** *A*, steady-state kinetics for mutant and wildtype variants of Fdc1 with cinnamic acid. *B–D*, pH dependence of the decarboxylation of cinnamic acid rate for wildtype and mutant forms of Fdc1. Concentration of E282D and E277D Fdc1 in assay: 600 and 300 nm, respectively.

The decarboxylation activity of the four Glu variants was tested using cinnamic acid as a substrate. Whereas the more conservative E282D variant was still able to catalyze decarboxylation of cinnamic acid, the corresponding E282Q variant was completely inactive, as shown by both end-point HPLC (Fig. 5) and UV-visible spectrophotometric activity assays. Where activity was detected, we determined the corresponding apparent values, as the relative occupancy of the prFMN^iminium^ might be subject to variation. For E282D, *k*_cat_^app^ was found to be 0.63 ± 0.04 s^−1^ and *K*_*m*_^app^ was 50 ± 10 μm, compared with the wildtype Fdc1 *k*_cat_^app^ of 9.4 ± 0.1 s^−1^ and *K*_*m*_^app^ of 10.0 ± 0.6 μm. Similarly, activity for E277D can be detected by both UV-visible decarboxylation assays and HPLC, with a *k*_cat_^app^ of 1.2 ± 0.2 s^−1^. An accurate value for *K_m_* could not be obtained for this variant, as it does not follow Michaelis–Menten kinetics (Fig. 6*A*). In contrast, only very low levels of decarboxylase activity could be detected for the E277Q variant by analyzing for product formation using HPLC, suggesting an upper limit for *k*_cat_^app^ of 0.3 min^−1^.

The effect of pH on the rate of decarboxylation by E282D and E277D was investigated (Fig. 6, *B–D*). In both cases, fitting the data to a bell curve reveals a pH optimum of 7, similar to the wildtype protein.

### Enzyme-catalyzed styrene H/D exchange confirms the need for acidic residues at 277 and 282

**Figure 7.**
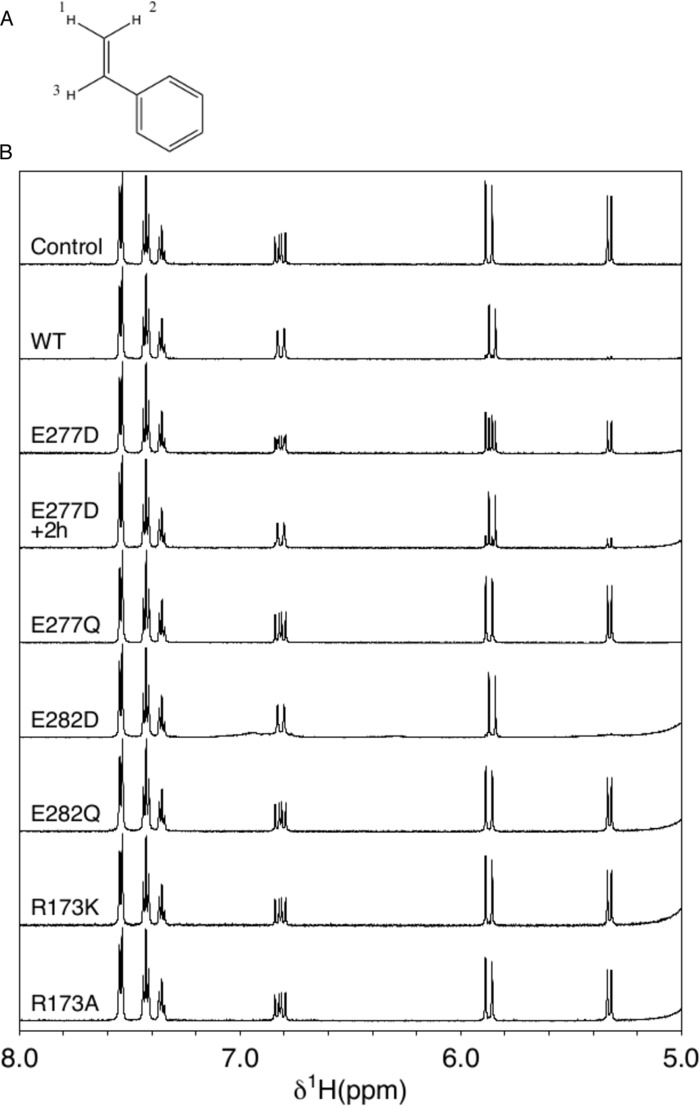
**H/D exchange of styrene.** Shown are ^1^H NMR spectra of styrene in D_2_O with and without the addition of wildtype and mutant variants of *A. niger* Fdc1. Enzyme-catalyzed deuterium exchange on the *trans*-position of C1 results in disappearance of resonance at 5.3 ppm and the simplification of the signal at 6.9 ppm from a doublet of doublets to a doublet. *Panel A* shows the individual styrene protons labeled according to corresponding NMR features shown in panel B, H1 related to 5.3, H2 related to 5.9, and H3 related to 6.9 ppm, respectively.

Hydrogen/deuterium exchange of styrene assessed using NMR spectroscopy was used to further examine the effect of the four Glu → Asp/Gln mutations on catalysis (Fig. 7*B*). Deuterium incorporation at the *trans*-position of styrene C1 (indicated by position 1 on Fig. 7*A*) indicates that proton abstraction of the substrate is able to occur (as a first step toward carboxylation) and that the cofactor must be in a catalytically active state. Disappearance of the resonance at 5.3 ppm, as well as simplification of the resonance at 6.9 ppm from a doublet of doublets to a doublet, indicates that exchange has taken place. Hydrogen/deuterium exchange occurs for wildtype and E282D within the 20-min dead time of the experiment. Exchange also occurs in the presence of the E277D variant, although much more slowly than observed with the wildtype protein; incubation for 2 h was required for ∼90% conversion. No significant exchange can be observed for E277Q, despite the detection of low levels of activity by HPLC. Similarly, H/D exchange was not observed for E282Q.

### Light-dependent cofactor isomerization is not dependent on the Glu–Arg–Glu motif

**Figure 8.**
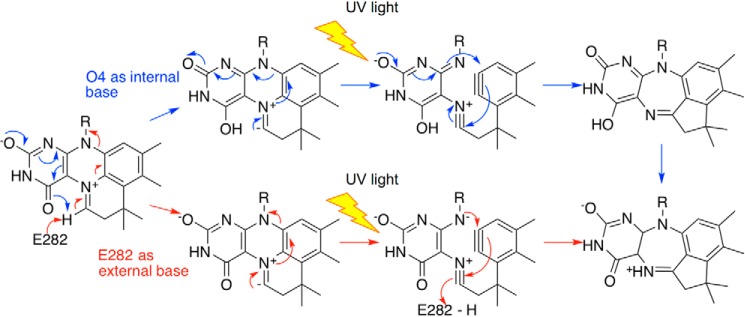
**UV-induced tautomerization of prFMN^iminium^ to prFMN^ketimine^.** Shown is a proposed scheme for the tautomerization reaction. *Blue arrows*, a mechanism whereby O4 acts as an internal base; *red arrows*, a mechanism whereby Glu^282^ acts as an external base in the first step.

The prFMN^iminium^ to prFMN^ketimine^ isomerization is likely to occur following deprotonation of the C1′ position, which could occur through either the adjacent prFMN O4 or through the Glu^282^ side chain (Fig. 8). To probe whether the enzyme active site is involved in this unusual isomerization, we tested the effect of light on key Fdc1 variants. Incubation of E282D and E277D (the only active variants) in the light leads to loss of activity over a time scale similar to that of wildtype, indicating that the formation of prFMN^ketimine^ is also possible in these variants (Fig. 2*A*). We furthermore determined the 1.28 Å crystal structure of E282Q (an inactive variant; see below) following UV light exposure (Fig. 2*F*), revealing that the prFMN^ketimine^ form can also be observed (albeit under 50% occupied) in the absence of the key catalytic acid-base residue.

### Arg^173^ variants reveal deficiencies in cofactor maturation

The UV-visible spectra of one of the purified Arg^173^ variants, R173A, exhibits a modest 550-nm spectral feature that has previously been associated with the prFMN^radical^ form of the cofactor (Fig. 3*C*). ESI-MS experiments using the R173A variant were able to detect MH^+^ cofactor masses of 525.16 and 526.16 Da, corresponding to the mature prFMN^iminium^ and prFMN^radical^ forms of the cofactor, respectively. In addition, a 541.16-Da species was also observed, which we attribute to prFMN^iminium^ having undergone subsequent hydroxylation and further oxidation (Fig. 4). HPLC activity measurements confirm that R173A retains low levels of activity as purified from cells co-expressing UbiX, confirming the presence of prFMN^iminium^ (Fig. 5).

**Figure 9.**
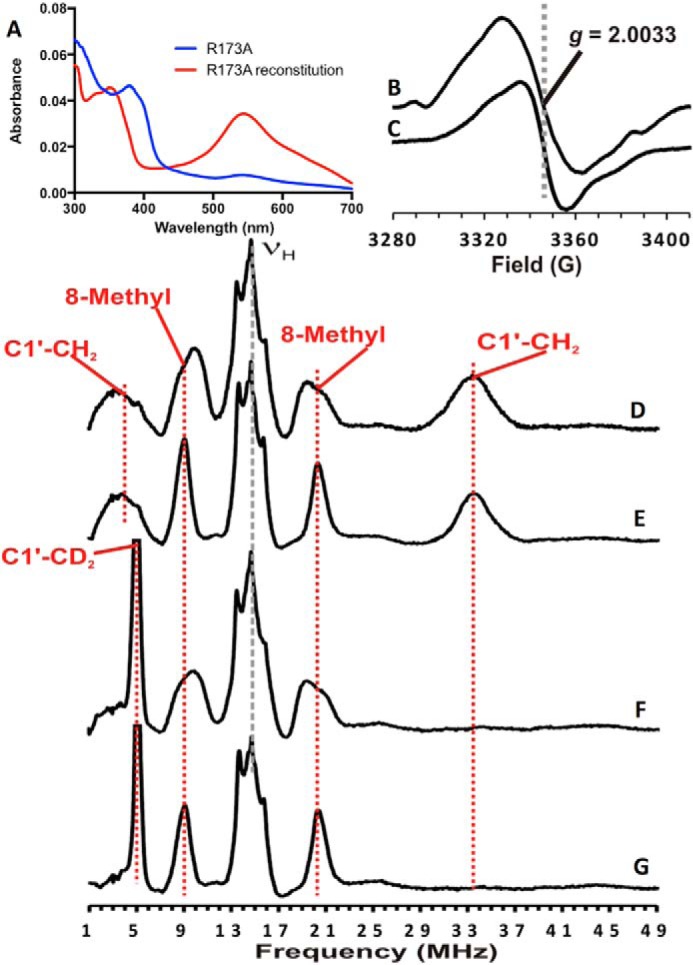
**UV-visible absorbance, EPR, and ENDOR spectra of prFMN-reconstituted Fdc1 R173A and as purified Fdc1 R173A.** *A*, UV-visible absorbance spectra of air-oxidized Fdc1 R173A, “as purified” (*blue*) and following reconstitution with prFMN^reduced^ (*red*); *B*, X-band EPR spectrum of the reconstituted and air-oxidized Fdc1 R173A; *C*, X-band EPR spectrum of air-oxidized Fdc1 R173A reconstituted with prFMN^reduced^ deuterated at C1′; *D*, the X-band Davies ENDOR spectrum of prFMN^radical^ formed in WT *E. coli* UbiD (7); *E*, the X-band Davies ENDOR spectrum of the Fdc1 R173A radical shown in *B*; *F*, the X-band Davies EPR spectrum of C1′-deuterated prFMN^radical^ formed in WT *E. coli* UbiD (7); *G*, the X-band Davies EPR spectrum of the Fdc1 R173A radical shown in *C*.

To further investigate cofactor maturation in R173A Fdc1, we undertook an *in vivo* reconstitution of this variant. The apo-R173A variant was obtained by expressing in the absence of UbiX. Apo-R173A was anaerobically reconstituted *in vitro* by adding prFMN^reduced^, produced by incubation of FMNH_2_ and DMAP with UbiX, to apo-R173A in an anaerobic environment, as reported previously (8). After the removal of excess prFMN, unreacted FMN, and DMAP, followed by oxidation via brief exposure to air, reconstituted R173A Fdc1 exhibited a UV-visible spectrum dominated by the 550-nm spectral feature (Fig. 9*A*), consistent with a large proportion of prFMN^radical^ being present. EPR spectroscopy also indicates the presence of the prFMN^radical^ (Fig. 9*B*) (6), with further support for this assignment arising from the narrowing of the EPR signal when deuterated DMAP was used in the biosynthesis of prFMN (Fig. 9*C*), as observed for *Escherichia coli* UbiD (7). Because such relatively featureless radical EPR signals can be difficult to assign to specific radicals, we also employed electron nuclear double resonance (ENDOR) spectroscopy, which provides a “fingerprint” for a radical through measurement of the hyperfine interactions between the unpaired electron of the radical and its constituent magnetic nuclei (in this instance hydrogen and deuterium atoms). The ENDOR spectra of air-oxidized reconstituted R173A Fdc1 are essentially identical to those that we reported previously for prFMN^radical^ in *E. coli* UbiD (Fig. 9, *D–G*) (7), confirming the assignment.

In contrast to R173A, the R173K variant did not appear to contain a significant amount prFMN^radical^, indicated by a very weak feature at 550 nm. Furthermore, no activity could be detected for R173K, despite the identification of an MH^+^ ion mass of 525.18 Da by ESI-MS (Fig. 4). H/D exchange experiments using styrene (Fig. 7*B*) confirmed limited activity for R173A, detectable at very low levels after incubation for 4 h, whereas no detectable exchange could be observed in the presence of R173K.

### Crystal structure of R173A confirms that cofactor maturation occurs over longer time scales

**Figure 10.**
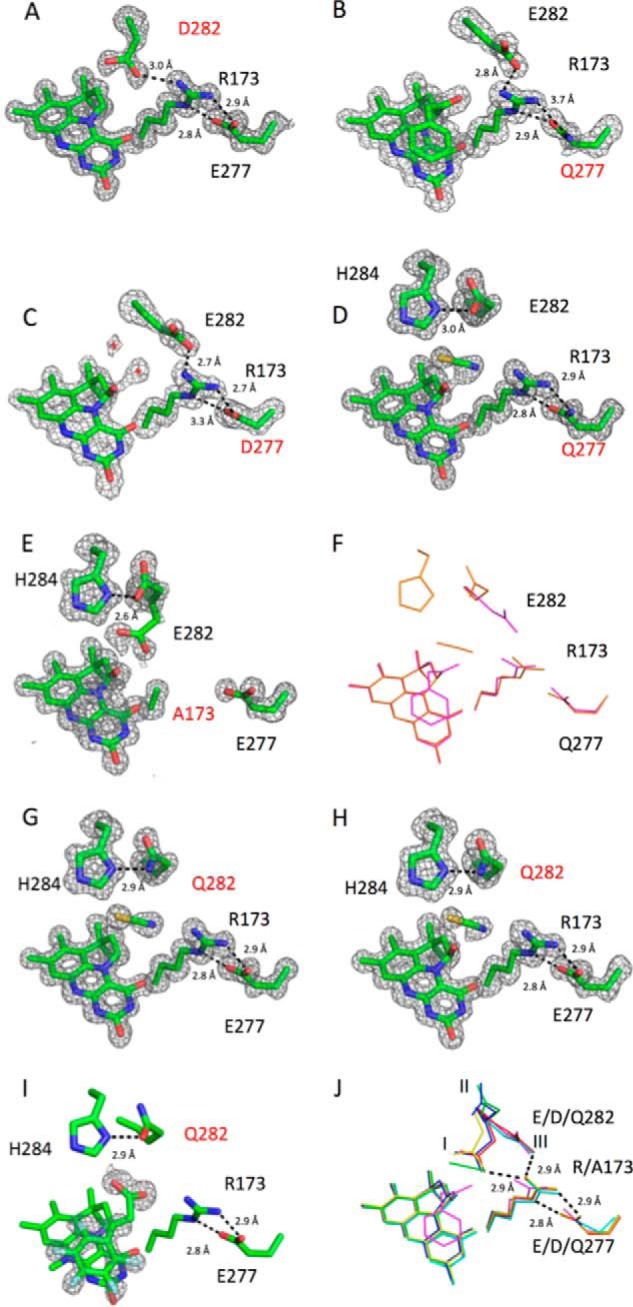
**Crystal structures of *A. niger* Fdc1 variants in complex with prFMN.** *A–E* and *G–H* show the active site in atom *colored sticks* for Fdc1 variants, with corresponding omit electron density contoured at 3σ. The mutated residues are *labeled* in *red* in each case. *F*, overlay of the two E277Q structures; the *purple sticks* correspond to the phenylpyruvate derived adduct (as in *B*), whereas the *orange sticks* correspond to the ligand-free structure reported in *D. I*, active site of the Glu^282^ variant in complex with pentafluorocinnamic acid, with the omit density corresponding to the substrate contoured to 3σ. *J*, overlay of the active-site structures of the five variants of *A. niger* Fdc1 with the wildtype structure (*red*, wildtype; *purple*, E277Q; *cyan*, E277D; *blue*, R173A; *green*, E282Q; *yellow*, E282D), with the three conformations adopted by residue Glu^282^ *labeled I*, *II*, and *III*. Hydrogen bonding is indicated by *black dotted lines*, and corresponding distances between key residues are shown for each *panel*; the distances shown for *J* correspond to wildtype structure.

The 1.19 Å crystal structure of R173A co-expressed with UbiX was obtained, revealing clear additional density on the prFMN C1′, confirming that hydroxylation readily occurred at that position (Fig. 10*E*). This suggests that the oxidative maturation of the cofactor did complete (before hydroxylation), albeit over long time scales. A similar observation was made for the *E. coli* UbiD enzyme, where a sulfite adduct was observed in the crystals (7).

In the R173A crystal structure, residue Glu^282^ has been modeled in two conformations, a prFMN-facing conformation (*labeled I* in Fig. 10*J*) and a second conformation that brings the carboxyl group of Glu^282^ to within hydrogen bonding distance (2.7 Å) of His^284^ (labeled II in Fig. 10*J*). In addition, there is a minor movement of residues 184–187, resulting in Leu^185^ moving away from the active site. These results, combined with the low levels of decarboxylase activity detectable by HPLC, and the small proportion of H/D exchange observed on styrene in the presence of R173A confirm that cofactor maturation can occur in the R173A variant. Unfortunately, repeated efforts to crystallize R173K were unsuccessful, so we cannot assess the effect of this mutation on the architecture of the active site.

### Crystal structures of Fdc1 Glu^282^ variants reveal minor structural variation

The high-resolution crystal structure for E282D (1.06 Å) reveals that residue Asp^282^ occupies position I (Fig. 10*A*), with slight movements in the protein backbone allowing the Asp^282^ carboxyl group to occupy a position similar to that of Glu^282^ in the wildtype enzyme (Fig. 10*J*). In this case, no C1′ adducts are visible in the electron density. All other residues occupy the same position as in the wildtype, with hydrogen-bonding distances remaining constant.

Residues Arg^173^ and Glu^277^ also occupy the same position as wildtype in the high-resolution crystal structures for E282Q (1.13 Å/1.28 Å) (Fig. 10, *G* and *H*). One structure shows the presence of a minor species corresponding to a prFMN hydroxylated at C1′ (Fig. 10*H*), whereas another structure, obtained from a separate protein preparation, shows unmodified prFMN (Fig. 10*G*). Residue Gln^282^ is observed in a conformation within hydrogen-bonding distance (2.9 Å) of His^284^ (position II). Additional density in the active site, in the position usually occupied by the carboxyl group of Glu^282^, has been interpreted as a molecule of thiocyanate (a component of the crystallization solution), mimicking the CO_2_ product. To assess whether the E282Q mutant prevents substrate binding, which could cause the lack of decarboxylase activity, we performed ligand soaks with pentafluorocinnamic acid. In the corresponding crystal structure, partial occupancy of pentafluorocinnamic acid could be observed, indicating that substrate binding remains possible (Fig. 10*I*).

### Crystal structures of Fdc1 Glu^277^ variants reveal significant C1′ adduct formation

The crystal structure of E277D (1.64 Å) also shows electron density corresponding to partial hydroxylation (∼30%) of the C1′ (Fig. 10*C*). Additional electron density located close to the C1′ could be representative of further modification of the C1′ at a very low occupancy. There is a slight shift in the protein backbone from residue 274 to 278 compared with wildtype; this allows the carboxyl group of Asp^277^ to occupy almost the same position as Glu^277^; however, a slight rotation of the acid group leads to an extension of one hydrogen bond from 2.8 to 3.3 Å (Fig. 10*C*). Residue Glu^282^ faces away from the active site and prFMN cofactor (*labeled III* in Fig. 10*J*).

Two crystal structures were obtained for E277Q, derived from two separate enzyme preparations. In the first structure (1.64 Å), extensive modification on the C1′ can be observed that is similar to the phenylpyruvate-derived inhibitor adduct observed for the WT enzyme (7) (Fig. 10*B*). It appears that under certain conditions, *E. coli* produces sufficient amounts of phenylpyruvate, leading to adduct formation *in vivo*, resulting in distinct enzyme preparations in the case of E277Q. In the E277Q phenylpyruvate adduct structure, the rotation of the Gln^277^ amide group and altered position of Arg^173^ disrupt the local hydrogen-bonding network between Gln^277^ and Arg^173^. The second crystal structure of E277Q (1.03 Å) only reveals partial hydroxylation of the C1′ (Fig. 10*D*). In the latter structure, the amide group of Gln^277^ occupies a similar position to the carboxyl of Glu^277^ in the wildtype protein (Fig. 10*J*). The presence of both of these adducts indicates that oxidative maturation of prFMN is able to take place for E277Q.

### Similar trends are observed for S. cerevisiae Fdc1 variants

**Figure 11.**
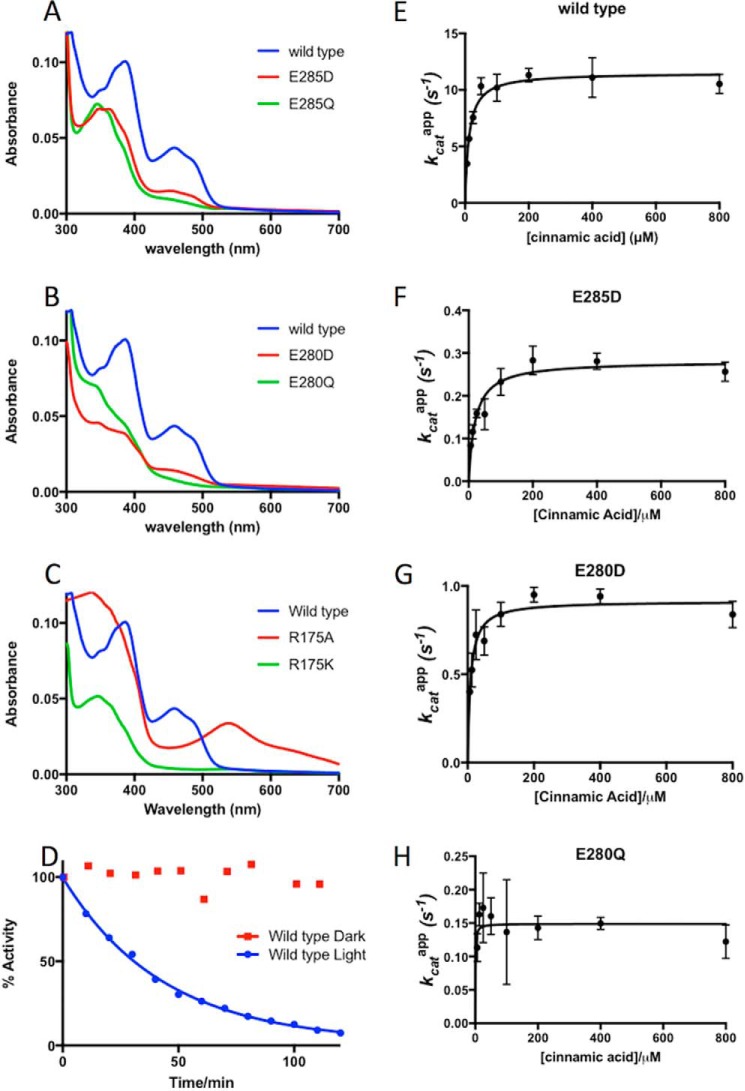
**UV-visible spectra and kinetic analysis for *S. cerevisiae* Fdc1 mutants.** *A–C*, UV-visible spectrum is shown for each variant of the three conserved active-site residues: Glu^285^ variants (*A*), Glu^280^ variants (*B*), and Arg^175^ variants (*C*). *D*, plot of activity over time for *S. cerevisiae* Fdc1 wildtype in the presence and absence of light exposure. *E–G*, steady-state kinetics for wildtype, E285D, E280D, and E280Q *S. cerevisiae* Fdc1 variants. Values are reported as apparent due to possible variations in the level of prFMN^iminium^ incorporation. Concentration of wildtype Fdc1 in the assay was 65 nm. Concentration of E285D, E280D, and E280Q Fdc1 in the assay was 630, 400, and 680 nm, respectively.

The corresponding six variants were also made in the related *S. cerevisiae* Fdc1, with the corresponding UV-visible spectra, decarboxylation assays (of cinnamic acid), and crystal structures revealing trends similar to those observed for the *A. niger* variants. Again, incubation of wildtype protein that has been co-expressed with UbiX on ice leads to a decrease in activity over time, with a half-life of ∼30 min (Fig. 11*D*); however, when incubated in the dark, activity remains stable for many hours. Although there is little evidence for prFMN^ketimine^ in any *S. cerevisiae* Fdc1 crystal structures, it is likely that the mechanism of inactivation is the same.

The UV-visible spectra for *S. cerevisiae* Fdc1 are complicated by the incorporation of unmodified flavin, indicated by the 450-nm peak visible in the wildtype spectrum. The *S. cerevisiae* Fdc1 variants studied bind a lower proportion of FMN, indicated by a reduction of the 450 nm peak, with features between 300 and 400 nm suggesting prFMN incorporation. Glu^280^ (equivalent to Glu^277^ of *A. niger* Fdc1) variants appear to bind the lowest proportion of unmodified flavin and exhibit different levels of prFMN binding, with differences in ∼340-nm centered features. As observed for *A. niger* Fdc1, the R175A mutant (*S. cerevisiae* numbering) shows a prominent 550-nm peak, indicating the presence of prFMN^radical^; however, this feature is absent in the R175K variant.

Decarboxylase activity can be detected by UV-visible spectrophotometric assay for the wildtype protein and E285D, E280D, and E280Q variants. Wildtype *S. cerevisiae* Fdc1 exhibits a *k*_cat_^app^ of 10.5 ± 0.4 s^−1^ and *K*_*m*_^app^ of 25.5 ± 3.6 μm. For E285D *k*_cat_^app^ has been measured as 0.28 ± 0.01 s^−1^, and *K*_*m*_^app^ has been measured as 20.4 ± 3.6 μm. For E280D, *k*_cat_^app^ has been measured as 0.92 ± 0.03 s^−1^, and *K*_*m*_^app^ has been measured as 8.6 ± 1.5 μm. For E280Q, *k*_cat_^app^ has been measured as 0.09 ± 0.02 s^−1^, and a value for *K_m_* could not be obtained (Fig. 6). Activity cannot be detected for R175A, which is consistent with the presence of prFMN^radical^ indicated by the UV-visible spectrum. Activity cannot be detected for R175K or E285Q despite there being no obvious 550-nm feature in these variants.

**Figure 12.**
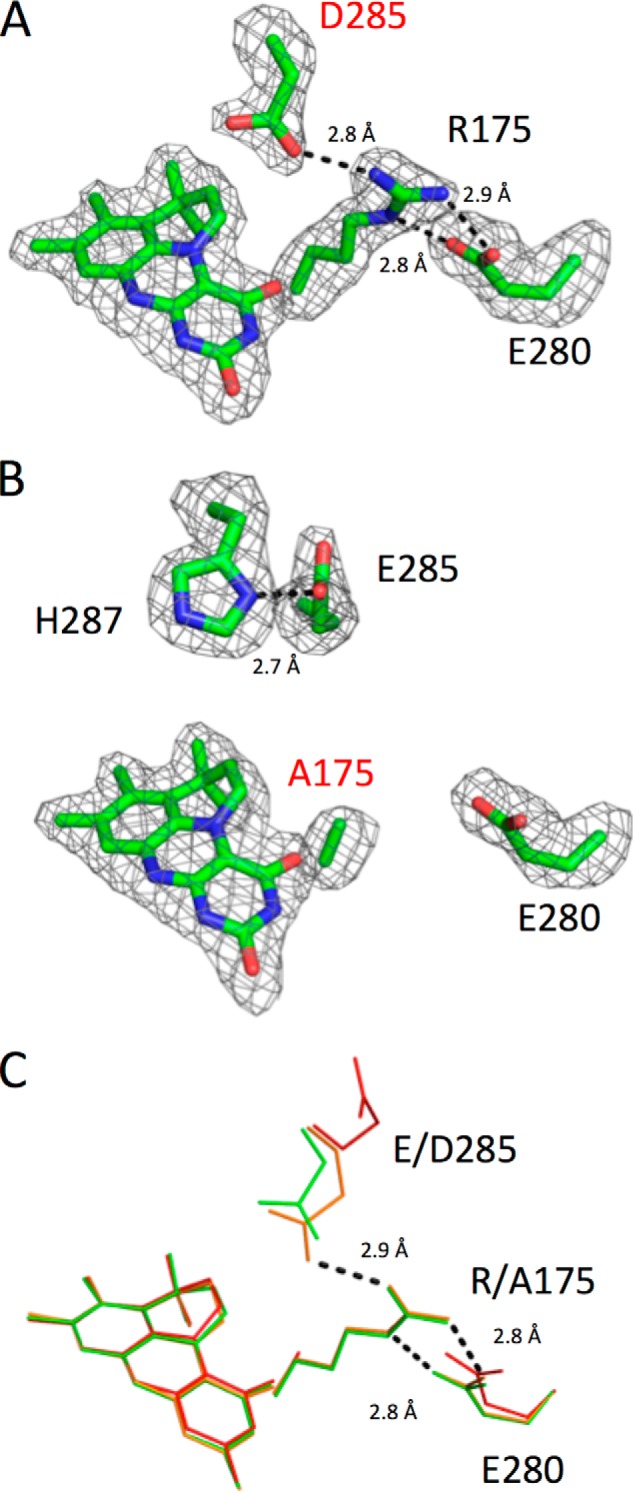
**Crystal structures of *S. cerevisiae* Fdc1 variants in complex with prFMN.** *A* and *B*, the prFMN and key residues in atom *colored sticks*. The corresponding omit electron density is shown contoured at 3σ. *C*, overlay of two Fdc1 variants (R175A in *red*, E285D in *green*) with the wildtype structure (*orange*). Key hydrogen-bonding distances between key residues are shown in each *panel*; the distances shown in *C* correspond to the wildtype structure.

Crystallization of wildtype, E285D, and R175A *S. cerevisiae* Fdc1 variants confirms cofactor incorporation (Fig. 12). E285D exhibits minimal disruption to the active-site architecture (Fig. 12, *A* and *C*), with hydrogen-bonding distances remaining very similar to those of wildtype and prFMN lacking modification on the C1′. The crystal structure of the R175A variant again shows prFMN in an unmodified form; here disruption of the ERE hydrogen bonding network has resulted in residue Glu^285^ moving away from the active site to within hydrogen-bonding distance (2.7 Å) of residue His^287^ (Fig. 12, *B* and *C*).

## Discussion

The UbiD family contains a highly conserved R*X_n_*E*X*_4_(E/D) sequence motif, which is believed to play a role in catalysis. However, in view of the UbiD cofactor requirement, there are in fact three individual processes in which the R*X_n_*E*X*_4_(E/D) motif could play a role that affects activity. These include the oxidative maturation of the cofactor, the light-induced isomerization of the iminium to the ketimine form, and acid-base catalysis during the (de)carboxylation by Fdc1.

Despite the fact that a mechanism for catalysis has been proposed for the prFMN^ketimine^ (7), indirect evidence supports the hypothesis that prFMN^iminium^ is the catalytically active form of the cofactor (7, 10, 11). The observation that light leads to irreversible inactivation of Fdc1 in solution and that illumination is associated with prFMN^ketimine^ formation *in crystallo* strongly suggests that prFMN^ketimine^ is not relevant to catalysis. The unusual isomerization from the prFMN^iminium^ to the prFMN^ketimine^ form is likely to occur following C1′ deprotonation. This particular step is similar to the prFMN oxidative maturation, where C1′ deprotonation and concomitant oxidation are required. The C1′ deprotonation could in principle occur with either the adjacent prFMN O4 acting as the base or the closely located Glu^282^ acting as the base, similar to what is proposed to occur during (de)carboxylation. As prFMN^ketimine^ can be observed in UV-illuminated E282Q crystals, it appears likely that O4 is able to act as an internal base, with no direct role for Glu^282^ in the light-dependent isomerization of prFMN.

This suggests that prFMN light-dependent isomerization might be a general feature of the prFMN^iminium^ form. However, we have only demonstrated light sensitivity in two Fdc1 enzymes, and light sensitivity has not previously been reported for other members of the UbiD superfamily. In fact, oxygen sensitivity has been reported for some members of the UbiD superfamily, such as 3,4-dihydroxybenzoate decarboxylase (8, 14), indole-3-carboxylate decarboxylase (15), and phthaloyl-CoA decarboxylase (16). It is possible that the latter enzymes might undergo a 1,3-dipolar cycloaddition with singlet oxygen, a potent dipolarophile that can be formed as a consequence of illumination (17). Further investigation will be required to determine whether this or another oxidative process is responsible for enzyme inactivation in these cases.

Similar to the isomerization process described above, maturation of prFMN^reduced^ to the catalytically relevant prFMN^iminium^ requires C1′ proton abstraction concomitant with oxidation. Previous studies have suggested that this process only occurs within the UbiD enzyme, suggesting a direct contribution of the enzyme to the oxidative process. Given the close proximity of Glu^282^, it is possible that this residue is required for C1′ proton abstraction. The E282Q variant of Fdc1 is inactive, which could be due to the inability to catalyze (de)carboxylation but could also result from defects in cofactor maturation. We reveal that crystal structures of E282Q do contain additional density on the C1′ of prFMN corresponding to partial formation of a hydroxyl adduct. This strongly suggests that prFMN^iminium^ is formed. Furthermore, the reduced prFMN is not stable in the presence of oxygen, rapidly forming a purple-colored radical species (6, 8). Both the E282Q variant in solution and crystals of E282Q remain colorless under aerobic conditions, suggestive of prFMN^iminium^ formation. Hence, Glu^282^ is not required for maturation of prFMN. Furthermore, we reveal that maturation is able to proceed in E282D, E277D, and E277Q, as indicated by enzyme activity, the identification of the ion mass corresponding to prFMN^iminium^ by ESI-MS in ED variants, and the formation of adducts on prFMN C1′ in corresponding crystal structures.

In contrast, both the UV-visible spectra of R173A and R173K variants exhibit a feature at 550 nm, characteristic of the presence of the purple semiquinone radical prFMN. Indeed, EPR experiments confirm the presence of this species in R173A. This suggests that oxidative maturation is perturbed in these variants, similar to what has been observed for the *E. coli* UbiD (8). Crystal structures of the R173A variant do contain a hydroxyl adduct on the prFMN C1′, whereas a mass corresponding to prFMN^iminium^ can also be detected for R173A, along with low levels of decarboxylase activity and H/D exchange. This indicates that oxidative maturation is affected in R173A, but can still occur. *In vitro* reconstitution of *apo*-R173A leads to the prFMN^radical^–Arg^173^ complex that appears stable over several hours, suggesting that further oxidation/maturation events are indeed extremely slow.

**Figure 13.**
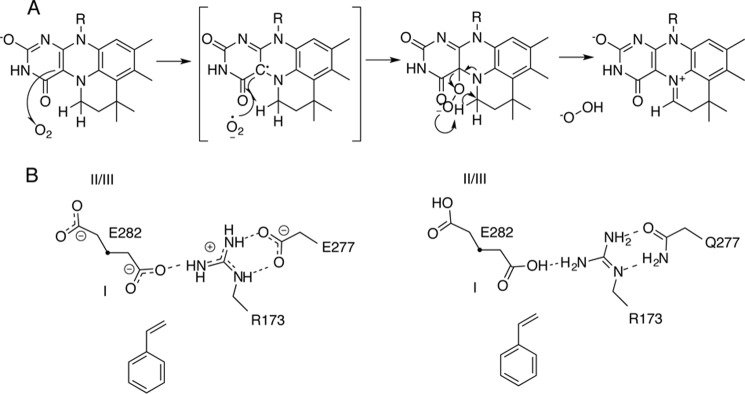
**Mechanistic proposal for prFMN oxidative maturation and the role of Glu^277^ in catalysis.** *A*, proposed mechanism for maturation of prFMN^reduced^ to prFMN^iminium^ in Fdc1. *B*, schematic showing the influence of residue 277 on the charge state of residues 173 and 282 in *A. niger* Fdc1. Distinct positions of residue Glu^282^ corresponding to the presence or absence of a substrate carboxylate group are *numbered I* and *III* and correspond to the *labels* in Fig. 10*J*.

Hence, cofactor maturation in Fdc1 does not require the presence of Glu^282^ but is affected by removal of the Arg^173^. However, the latter is located too far away from C1′ to be directly involved in proton abstraction. Instead, we propose that prFMN oxidation by oxygen proceeds in a manner similar to that observed for flavins, with transfer of an electron to O_2_, leading to formation of the observed semiquinone prFMN^radical^ and superoxide, as shown in Fig. 13. The loss of the superoxide would lead to a trapped semiquinone species, as observed in UbiX (6), *E. coli* UbiD (8), and Fdc1 R173A. Rapid recombination of the superoxide and prFMN^radical^ leads to a C4a peroxoadduct, which can act to abstract a proton from C1′ during elimination of peroxide. A similar C4a peroxoadduct has been synthesized *in vitro* by the addition of hydrogen peroxide to *N*^5^-alkylated FMN (18). As the Arg^173^ side chain is probably positioned close to the postulated C4a peroxo moiety, mutation of this residue is likely to affect its formation as well as associated acid-base properties.

Thus, Glu^282^ appears only to play a role in catalysis, where it is postulated to act as acid-base during (de)carboxylation of the substrate–cofactor complex. In fact, a proportion of the UbiD family have an Asp at the position corresponding to Glu^282^, including the canonical UbiD enzyme. The requirement for an acidic residue at this position strongly suggests that there is an enzyme-mediated proton transfer step in the catalytic mechanism. Residue Glu^282^ is seen in three distinct positions, as indicated in Fig. 10*J*. Movement of residue 282 during the catalytic cycle of Fdc1 could therefore facilitate the shuttling of protons between solvent and the active site. Both the E282Q (*A. niger*) and E285Q (*S. cerevisiae*) variants are inactive, whereas E282D and E285D variants retain a considerable level of decarboxylase activity. Furthermore, the pH dependence of the reaction for both Glu → Asp variants is similar to that observed for the WT (Fig. 6). This suggests the p*K_a_* of key groups involved in catalysis is unperturbed by the Glu → Asp substitution, with the lower *k*_cat_ values observed for E282D and/or E285D possibly resulting from increased proton transfer distances, as the carboxylate group is slightly shifted in crystal structures of these variants. Surprisingly, activity for the E277Q variant was very low, although crystal structures reveal no significant structural perturbation in the active site. This suggests that Glu^277^ acts to tune the p*K_a_* of Arg^173^ and in turn Glu^282^. Unfortunately, due to the extremely low activity, it was not possible to accurately measure the pH dependence of the reaction. The negative charge provided by Glu/Asp^277^ is likely to increase the Arg^173^ p*K_a_*, in turn lowering the Glu^282^ p*K_a_* (Fig. 13*B*). Furthermore, given the fact that the acid substrate binds adjacent to Arg^173^, the Arg^173^–Glu^277^ duad is responsible for selective binding and positioning of the deprotonated substrate.

In conclusion, the UbiD R*X_n_*E*X*_4_(E/D) motif is required for efficient catalysis, but only the Arg^173^ position appears to influence cofactor maturation. The Glu(Asp)^282^ acts as the key acid-base, with the Arg^173^–Glu^277^ duad playing a role in fine-tuning the Glu(Asp)^282^ properties as well as substrate binding. The exact mechanism for oxidative maturation remains unclear, but it seems plausible that this resembles the mechanism for flavin oxidation, with Arg^173^ influencing the formation and properties of the C4a peroxoadduct. The unusual and irreversible isomerization of prFMN^iminium^ to prFMN^ketimine^ is light-dependent and probably proceeds via high-energy intermediates. Whereas this does not appear dependent on the R*X_n_*E*X*_4_(E/D) motif, it is unclear to what extent prFMN^ketimine^ formation might affect other UbiD family members. Flavins have been used to underpin light-sensing systems in nature (17), and it is possible that the prFMN light-dependent isomerization might feature in a biological process.

## Experimental procedures

### Cloning

The *A. niger fdc* gene was codon-optimized and synthesized (Genescript). The *A. niger fdc1* gene was cloned into the NdeI and XhoI sites of pET30a, and the *S. cerevisiae fdc1* gene was cloned into the NdeI and XhoI sites of pET21b. *E. coli ubiX* was cloned into the NdeI and XhoI sites of pET21b and pET30a. *A. niger fdc1* pET30a was transformed into *E. coli* BL21(DE3) with and without *E. coli ubiX* pET21b. *S. cerevisiae fdc1* pET21b was transformed into *E. coli* BL21(DE3) with and without *E. coli ubiX* pET30a.

### Mutagenesis

Mutagenesis primers were designed using the QuikChange primer design program. PCR was performed using Phusion Polymerase (New England Biolabs). Template was digested using DpnI, and the PCR product was transformed into *E. coli* NEB5α. The presence of the desired mutation was confirmed by sequencing (Eurofins). The plasmid was then co-transformed with the corresponding UbiX construct into *E. coli* BL21 (DE3).

### Protein expression

Protein was expressed in *E. coli* BL21 (DE3) grown in LB medium, supplemented with 50 μg ml^−1^ ampicillin and 50 μg ml^−1^ kanamycin at 37 °C, 180 rpm until mid-log phase. The cultures were then cooled to 15 °C, supplemented with 1 mm MnCl_2_, induced using 0.25 mm isopropyl 1-thio-β-d-galactopyranoside (Formedium), and grown overnight. Cells were harvested by centrifugation (4 °C, 7,000 × *g*, 10 min).

### Purification of A. niger Fdc1

Cell pellets were resuspended in Buffer A (50 mm Tris, 200 mm NaCl, pH 7.5, in Milli-Q water) supplemented with complete EDTA-free protease inhibitor mixture (Roche Applied Science), lysozyme, DNase, and RNase (Sigma). The cells were lysed on ice using a Bandelin Sonoplus sonicator with a TT13/F2 tip, set to 30% power, 20 s on, 40 s off for 30 min. Cellular debris was removed by ultracentrifugation using a Ti50.2 rotor in a Beckman Optima LE-80k ultracentrifuge at 40,000 rpm for 1 h at 4 °C. The supernatant was passed through a 0.45-μm filter. The clarified supernatant was applied to a gravity flow nickel-nitrilotriacetic acid-agarose column (Qiagen). The column was washed with three column volumes of Buffer A supplemented with 10 mm imidazole and then three column volumes of Buffer A supplemented with 40 mm imidazole. Protein was eluted in 1-ml fractions using Buffer A supplemented with 250 mm imidazole. Fractions containing the purified protein were buffer-exchanged into Buffer B (20 mm Tris, 100 mm NaCl, pH 7.5, in Milli-Q water) using a 10DG column (Bio-Rad). Protein aliquots were flash-frozen until required.

### Purification of S. cerevisiae Fdc1

Purification of *S. cerevisiae* Fdc1 was as above, using 50 mm KPO_4_, 200 mm NaCl as Buffer A and 20 mm KPO_4_, 100 mm NaCl as Buffer B.

### In vitro reconstitution of apo-Fdc1

R173A Fdc1 was reconstituted *in vitro* using prFMN produced by *P. aeruginosa* UbiX as described previously (8).

### UV-visible spectroscopy and protein quantification

UV-visible absorbance spectra were recorded using a Cary 50 Bio spectrophotometer (Varian). Protein concentrations were calculated using ϵ_280 nm_ = 63,830 m^−1^ cm^−1^ for *A. niger* Fdc1 variants and ϵ_280 nm_ = 68,870 m^−1^ cm^−1^ for *S. cerevisiae* variants. All spectra have been normalized for protein content.

### UV-visible spectrophotometric decarboxylation assays

The initial rate of decarboxylation was determined by following consumption of substrate by UV-visible spectroscopy using a Cary 50 Bio spectrophotometer (Varian). Assays were performed against various concentrations of substrate in 350 μl of 50 mm KCl, 50 mm NaP_i_, pH 6, in a 1-mm path length cuvette at 25 °C. The rate of cinnamic acid consumption was measured at 270 nm. The extinction coefficient for cinnamic acid is ϵ_270 nm_ = 20,000 m^−1^ cm^−1^, and the extinction coefficient for styrene is ϵ_270 nm_ = 200 m^−1^ cm^−1^. The rate of cinnamic acid consumption was calculated using Δϵ_270 nm_. Protein concentration was determined using *A*_280_, and all *k*_cat_ values are apparent due to variations in prFMN content. The final concentration of protein in each assay was varied to ensure that measurements of cinnamic acid consumption could be taken within the linear portion of the experiment.

### Light/dark studies

Time courses to compare loss of activity in the light and dark were carried out by incubating protein in either clear or black plastic microcentrifuge tubes, with measurements of decarboxylase activity carried out as above. The UV-visible spectrum of UV-treated Fdc1 was acquired by exposing protein to UV light from a 365-nm LED source (Thorlabs).

### Hydrogen/deuterium exchange assays

An excess of styrene was mixed with 50 mm KP_i_, pH 6, in D_2_O to obtain a saturated solution of styrene in D_2_O (∼3%, v/v). After the addition of protein to a final concentration of 35 μm, the ^1^H NMR spectra were recorded at 298 K on a Bruker 600-MHz AVI NMR spectrometer with a TXI cryoprobe equipped with *z*-gradients, using presaturation for water signal suppression (1.7-s acquisition time, 2-s interscan delay, 90° ^1^H pulses). Dead time between enzyme addition and recording of spectra was ∼20 min, except where indicated. Chemical shifts were referenced to trimethylsilylpropionic acid.

### HPLC decarboxylation assays

Assays containing 10 mm cinnamic acid, 50 mm KCl, 50 mm NaP_i_, pH 6, were incubated for 2 h at 25 °C with 50 μm enzyme. 100 μl of sample was added to 900 μl of 50% (v/v) H_2_O/acetonitrile with 0.1% TFA. Sample analysis was performed using an Agilent 1100 series HPLC equipped with a UV detector. The stationary phase was a Kinetex 5-μm C18 column, 250 × 4.6 mm. The mobile phase was acetonitrile/H_2_O (50:50), with 0.1% TFA. Detection was performed at 254 nm.

### Protein crystallization and structure determination

Crystallization was performed by sitting-drop vapor diffusion. An initial screening of 0.3 μl of 14 mg ml^−1^ *A. niger* Fdc1 in 100 mm NaCl, 25 mm Tris, pH 7.5, and 0.3 μl of reservoir solution at 4 °C resulted in a number of hits, including PACT condition F4 (Molecular Dimensions). Seed stock produced from these crystals was used in an optimization screen based around 0.2 m potassium thiocyanate, Bistris propane, pH 6.5, 20% (w/v) PEG 3350 by mixing 0.05 μl of seed stock, 0.25 μl of protein solution, and 0.3 μl of reservoir solution at 4 °C. For *S. cerevisiae* Fdc1, an optimization screen was developed based around CSII condition C6 (Molecular Dimensions), 0.1 m sodium cacodylate, pH 6.5, 0.25 m calcium acetate, 15% PEG 4000. Crystals were obtained by mixing 0.05 μl of seed stock, 0.25 μl of protein solution, and 0.3 μl of reservoir solution at 4 °C. Crystals were cryoprotected in reservoir solution supplemented with 10% PEG 200 and flash-cooled in liquid nitrogen. Diffraction data (Table 1) were collected at Diamond beamlines and processed using the CCP4 suite (19). Molecular replacement was undertaken in Phaser MR (20) using 4ZA4 as a model; further refinement was carried out using REFMAC5 (21) and manual rebuilding in COOT (22). Ligand coordinates and definitions were generated using AceDRG (23).

**Table 1:**
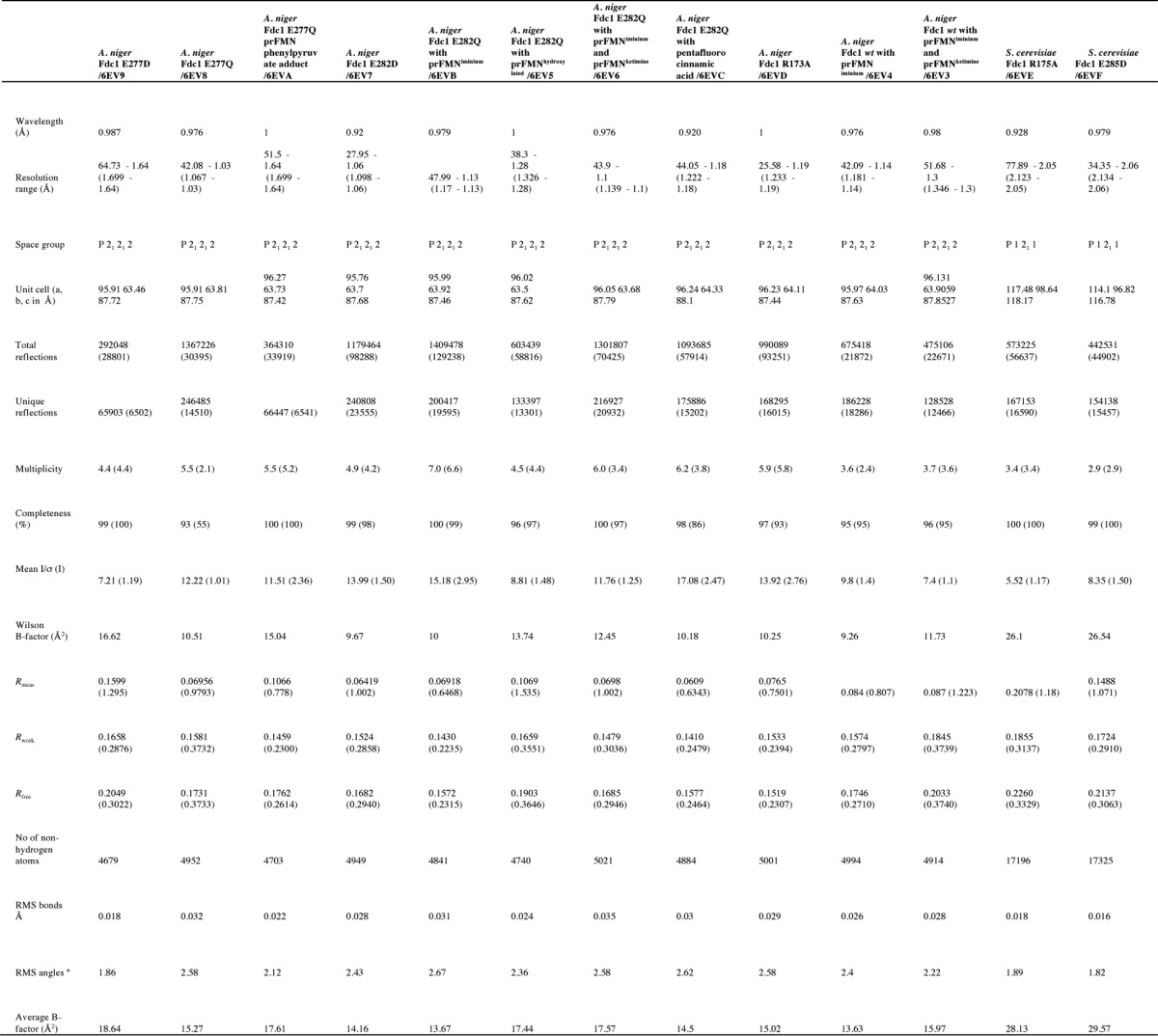
**Crystallographic data collection and refinement statistics**

*^a^* Values for the highest-resolution shell are shown in parentheses.

*^b^* RMSD, root mean square deviation.

### EPR and ENDOR spectroscopy

EPR and ENDOR spectra were obtained using a Bruker E500/580 EPR spectrometer. Continuous wave X-band EPR spectra employed a Bruker “Super High Q” cavity (ER 4122SHQE) coupled to an Oxford Instruments ESR900 helium flow cryostat for temperature control. Spectra were acquired at 20 K using 10-microwatt microwave power, 100-kHz field modulation frequency, and 1 G modulation amplitude. X-band FID-detected Davies pulsed ENDOR spectra were collected at 30 K and *g* = 2.0033 using a Bruker EN 4118X-MD4 dielectric ENDOR resonator coupled to an ER 4118HV-CF100 Cryo-Free cooling system. The length of the initial inversion pulse was 400 ns, the detection pulse was 200 ns, and the radiofrequency pulse was 9 μs.

### Mass spectrometry

Samples were prepared by a desalting of proteins into 100 mm ammonium acetate, pH 7.0. A 1200 series Agilent LC was used to inject 5 μl of sample into 5% acetonitrile (0.1% formic acid) and desalted inline to release the cofactor from the enzyme complex. This was eluted over 1 min by 95% acetonitrile. The resulting ions were analyzed by an Agilent QTOF 6510 run in positive mode and deconvoluted using Agilent Masshunter software.

## Author contributions

S.S.B., K.A.P.P., K.F., M.J.C., R.S., S.E.J.R., and D.L. formal analysis; S.S.B., K.A.P.P., K.F., and D.L. validation; S.S.B., K.F., S.A.M., and D.L. investigation; S.S.B. visualization; S.S.B., K.A.P.P., S.A.M., and S.E.J.R. methodology; S.S.B. and D.L. writing-original draft; S.S.B., K.A.P.P., K.F., S.A.M., M.J.C., R.S., D.A.P., S.E.J.R., and D.L. writing-review and editing; M.J.C., R.S., S.E.J.R., and D.L. data curation; D.A.P. resources; D.A.P. and D.L. funding acquisition; D.A.P. and D.L. project administration; S.E.J.R. and D.L. supervision; D.L. conceptualization.

## Footnotes

2 The abbreviations used are:

prFMN: prenylated flavin mononucleotide
Bistris propane: 1,3-bis[tris(hydroxymethyl)methylamino]propane
ESI: electrospray ionization
H/D: hydrogen/deuterium
ENDOR: electron nuclear double resonance
DMAP: dimethylallyl phosphate.
